# Sham controls in device trials for chronic pain – tricky in practice-a review article

**DOI:** 10.1016/j.conctc.2023.101203

**Published:** 2023-08-22

**Authors:** Selina Johnson, Andreas Goebel

**Affiliations:** a Walton Centre NHS Foundation Trust, Fazakerley, Liverpool, L9 7BB, UK; b Pain Research Institute, Institute of Life Course and Medical Sciences, Musculoskeletal and Ageing Science, University of Liverpool, Fazakerley, Liverpool, L9 7AL, UK

**Keywords:** Sham control, Device, Review, Chronic pain, Conduct, methods

## Abstract

**Background:**

Chronic pain affects one in four people and this figure is likely to increase further in line with an ageing population. Efforts to evaluate nonpharmacological interventions to support this patient population have become a priority for pain research. For device trials, the use of a sham control can add to the scientific validity and quality of a study. However, only a small proportion of pain trials include a sham control, and many are of poor quality. To facilitate the conduct of high-quality trials there is a need for a comprehensive overview to guide researchers within this area. The objective of this review was to synthesise the published data to address this need.

**Methods:**

We identified studies that considered the evaluation, design, and conduct of sham-controlled trials in chronic pain by searching MEDLINE, CINAHL and Science Direct to November 2022. Studies that included sufficient content to inform the conduct/design of future research were included. An inductive thematic analysis approach was used to identify themes that require consideration when conducting sham-controlled trials. These are presented as a narrative review.

**Results:**

37 articles were included. Identified themes related to the type of sham device, sham design, bias, study population and ethics.

**Conclusions:**

To conduct good quality research the challenges surrounding the use of sham interventions need to be better considered. We highlight salient issues and provide recommendations for the conduct and reporting of sham-controlled device trials in chronic pain.

## 1 Background

Chronic pain (CP) is estimated to affect one in four people in the UK [1]. Guidelines acknowledge that medications and surgery have limited value for the management of ongoing CP [2]. As such there is a growing interest in medical devices that may support the management of CP. For device trials, high-quality evidence is required to limit the potential harms associated with patients being exposed to ineffective treatments and evidence-based care. Randomised controlled trials (RCTs) are considered the *gold standard* in research in terms of demonstrating treatment efficacy and producing high-quality evidence [3]. The most rigorous type of RCT is a ‘double-blind RCT’, where clinicians and participants are unaware of the treatment received. Double blinding further reduces bias, such as unspecific effects arising from the knowledge of receiving a presumably active intervention [4]. A sham control describes a procedure/intervention designed to resemble the procedure/intervention being tested but that does not contain the component thought to be associated with a therapeutic effect [5,6]. As such a sham control can be used as a comparator to an active treatment to facilitate the conduct of double-blind device RCTs [5]. Sham controls could, therefore, facilitate the conduct of high-quality evidence free of many forms of bias. However, a recent search of the Medline database revealed that of 8233 interventional CP studies only 340 (4%) employed a sham control [7]. One suggested reason for the low number of such trials is the understanding that the development of credible sham procedures/interventions is often challenging [8]. Initial scoping searches of the literature found that guidance surrounding the conduct of sham-controlled device trials does not describe multiple themes and issues relevant to this area. Therefore, there is a need for a comprehensive overview that synthesises multiple themes to guide researchers and promote the conduct of high-quality future research.

## 2 Methods

A narrative review was conducted as part of PhD thesis [9], to answer the research question 'What are the key issues and considerations in justifying the use of and designing sham-controlled device trials for chronic pain?'.

Aim: To identify major themes and considerations relevant to the conduct of sham-controlled pain trials.

### 2.1 Search strategy

To address this the PICO method (Table 1) was used to formulate a search strategy (Table 2) to identify studies that considered the evaluation, design, and conduct of sham-controlled trials in chronic pain. The world health organisation medical device definition *'Any instrument …. machine, appliance, implant, ….intended for a medical purpose'* [10], was used to identify intervention search terms. Outcome search terms were chosen to capture information relating to and informing the conduct and design of trials. Further references were identified via hand searching of study references. MEDLINE, CINAHL and Science Direct databases were searched to January 2019 and re-ran in November 2022. Searches were limited to English language, human subjects, and peer-reviewed publications.

**Table 1:** A PICO table illustrating the review criteria.

Population	Chronic pain
**I**ntervention	Any device, machine, appliance, implant or intervention intended for use as a sham.
**C**omparator	Any comparator or no comparator
**O**utcome	Evaluation, issue, design, consideration, problem, and conduct.

**Table 2:** Example search strategy.

Databases searched	
Search strategy	**Search Strategy – Medline (Ovid)** 1 Exp chronic pain/ 2 Sham 3. control 4. device 5. machine 6. appliance 7. implant 8. intervent* 9. intervention* 10. 3 or 4 or 5 or 6 or 7 or 8 or 9 11. 1 and 2 and 10 12. evaluat* 13. issue* 14. design 15. consideration* 16. problem* 17. conduct 18. 12 or 13 or 14 or 15 or 16 or 17 19. 11 and 18 Limit to (English language and humans)
* Denote where truncation was used as part of searches.	

Table adapted from Johnson [9] (table 4.1 page 79).

#### 2.1.1 Study eligibility

All references underwent a title and abstract screening stage before proceeding to the full-text review. The study eligibility screening tool (Table 3) was used to screen studies with respect to inclusion and exclusion criteria. Studies were included if they considered the evaluation, conduct or design of device, machine, appliance, or implant interventions. All types of study were considered. Studies were excluded if they referred to animal studies, acute pain, or included pharmacological or surgical apparatus interventions. Studies were additionally excluded if in the opinion of the authors they failed to include sufficient content to inform the conduct of future research. All abstracts and full texts were reviewed by one author and checked for consistency by the second author.

**Table 3:** Inclusion criteria screening tool.

	Include	Exclude
Population	F07F Chronic pain	F07F Acute pain defined <6 months duration. F07F Animal studies
Intervention	F07F Sham control- defined as any device/intervention intended as a sham control. This could include. o device, o machine, o appliance, o implant o intervention	F07F pharmacological agent F07F surgical apparatus
Comparator	F07F Any or none	F07F n/a
Outcomes	F07F outcome terms that inform the conduct of sham-controlled trials these may include: o Evaluation, o issue, o design, o consideration, o problem, o conduct.	F07F In the opinion of the reviewer the description of sham control would not inform the conduct/design of future research.
Study design	F07F All study types	F07F Studies only available in the abstract form were excluded due to insufficient detail to inform narrative review.
Language	F07F English	F07F Non-English
Overall decision	F07F INCLUDED	F07F EXCLUDED

#### 2.1.2 Data extraction, analysis, and synthesis

Data extraction included study author, year, study type, study area, main themes, subthemes, and conclusions.

An adapted inductive thematic analysis approach was used to identify themes [11]. This involved the following stages: 1) Source identification, 2) Familiarisation, 3) Coding, 4) Identification of initial themes, 5) Reviewing themes to identify broader themes and subthemes, 6) Defining and naming themes, 7) Writing up in a logical narrative sequence, including why it is important to the broader study question (Table 4). Where similar articles by the same author(s) were identified only one paper is referred to.

**Table 4:** Identified themes relevant to sham trial design and conduct.

Main Identified Themes	Sub- Themes
Type of sham device	No perceivable output
	Sub-therapeutic dosing: • Shorter treatment duration • Lower strength stimulation
Design issues	Mechanism of action
	Validation
Sham- controlled trials and bias	Blinding
	Assessment of blinding
	Clinical interactions
	Expectation
Study population	Placebo effects
Ethics	Equipoise
	Risk-benefit balance
	Informed consent
	Deliberate deception

Table adapted from Johnson [9] (table 4.2 page 81).

## 3 Results

**Fig. 1:**
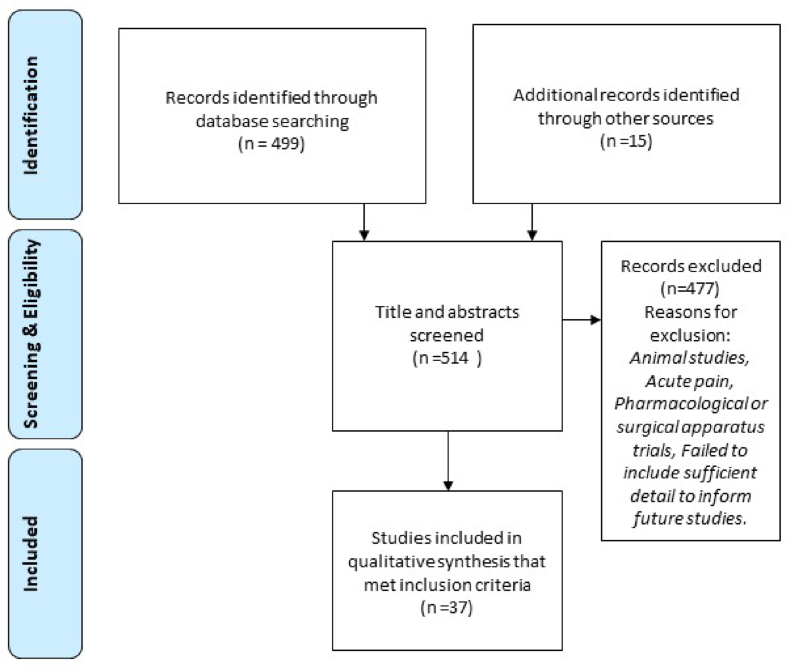
PRISMA flow chart Figure adapted from Johnson [9] (Figure 4.1 page 80).

Initial searches of electronic databases returned 497 records and 15 additional articles via article references (total 512); a rerun of searches in Nov 2022 identified a further 2 references (total 514), 477 of these abstracts were excluded (Fig. 1), leaving 37 articles informing the narrative review (Table 5).

**Table 5:** Description of studies included in the review and main themes.

	Article reference	Study Type	Study area	Main themes	Subthemes
1	Brim & Franklin [6] 2013	Position paper	The benefit of the placebo effect in sham-controlled trials	Ethics	Risk-benefit balance, informed consent
2	Katz et al. [8], 2021	Consensus recommendations	Research design considerations for randomized controlled trials of spinal cord stimulation for pain	Mitigating bias in sham-controlled trials	Blinding
3	Dworkin et al. [15], 2010	Consensus recommendations	Research design considerations for confirmatory chronic pain clinical trials	Type of sham	No perceivable output
4	Raphael et al. [12], 2011	Sham- RCT	Percutaneous Electrical Nerve Stimulation in Neuropathic Pain	Type of sham	No perceivable input
				Design issues relating to sham	Validating the intended sham device
5	Ghoname et al. [13] 1999	Sham- RCT	Percutaneous electrical nerve stimulation for low back pain	Type of sham	No perceivable output
6	Hamza et al. [14], 2000	Sham- RCT	Percutaneous electrical nerve stimulation for diabetic neuropathy	Type of sham	No perceivable output
				Design issues relating to sham	Validating the intended sham device
7	Brunoni et al. [16] 2012	Systematic review	Transcranial Direct Current Stimulation (tDCS) research recommendations	Type of sham	No perceivable output
8	White et al. [17], 2001	Consensus recommendations	Recommendations for optimal treatment, sham controls and blinding of Acupuncture research.	Type of sham	No perceivable output
9	Boutron et al. [18] 2007	Systematic review	Reporting methods of blinding in randomized trials assessing nonpharmacological treatments	Type of sham	No perceivable output
				Mitigating bias in sham-controlled trials	Blinding, assessment of blinding
10	Gibson et al. [19], 2017	Systematic review	Transcutaneous nerve stimulation for neuropathic pain	Type of sham	Subtherapeutic dosing
				Mitigating bias in sham-controlled trials	Blinding
11	Duarte et al. [21], 2020	A Systematic Review and Methodological Appraisal	Randomized Placebo-/Sham-Controlled Trials of Spinal Cord Stimulation	Type of sham	Subtherapeutic dosing
				Mitigating bias in sham-controlled trials	Blinding
12	Hoffman et al. [22], 2014	Consensus recommendations	Reporting of interventions: Template for intervention description and replication (TIDieR)	Mitigating bias in sham-controlled trials-	Blinding, Clinical interactions
				Design issues relating to the sham	Mechanism of action
13	Birch et al. [27], 2022	Literature review	Historical perspectives on using sham acupuncture in acupuncture clinical trials	Design issues relating to the sham	Validating the intended sham device
14	Kim et al. [28], 2022	Systematic review	Plausible mechanism of Sham Acupuncture Based on Biomarkers	Design issues relating to the sham	Validating the intended sham device
15	Sheffer et al. [29], 2013	Single blind study	Evaluation of sham repetitive transcranial Direct Current Stimulation	Design issues relating to sham	Validating the intended sham device
16	Vetter et al. [31], 2017	Topical review	Bias, Confounding, and Interaction in research	Mitigating bias in sham-controlled trials	Blinding
17	Haahr et al. [32], 2006	Cohort study	Who is blinded in randomized clinical trials? A study of 200 trials and a survey of authors.	Mitigating bias in sham-controlled trials	Blinding
18	Hróbjartsson et al. [33] 2007	Cohort study	An analysis of randomized clinical trials that report tests for the success of blinding.	Mitigating bias in sham-controlled trials	Blinding, assessment of blinding
19	Higgins et al. [35], 2011	Consensus recommendation and bias tool	Risk of bias tool for RCTs	Mitigating bias in sham-controlled trials	Blinding, assessment of blinding
20	Boutron et al. [36], 2010	Systematic review	Reporting and interpretation of randomized controlled trials with statistically nonsignificant results for primary outcomes	Mitigating bias in sham-controlled trials	Blinding
21	Carroll et al. [37], 2000	Systematic review	Transcutaneous nerve stimulation for neuropathic pain	Mitigating bias in sham-controlled trials	Blinding
22	Sterne et al. [38], 2019	Consensus recommendation and bias tool	Risk of bias tool for RCTs	Mitigating bias in sham-controlled trials	Blinding, assessment of blinding
23	Chen et al. [39], 2019	Clinical trial	Socially transmitted placebo effects	Mitigating bias in sham-controlled trials	Clinical interactions
24	Di Blasi et al. [40], 2001	Systematic review	Influence of context effects on health outcomes	Mitigating bias in sham-controlled trials	Clinical interactions
25	Rief et al. [41], 2012	Randomised experimental study	The hidden effects of blinded, placebo-controlled randomized trials.	Mitigating bias in sham-controlled trials	Clinical interactions
26	Laferton et al. [44], 2017	Review	Patients' Expectations Regarding Medical Treatment	Mitigating bias in sham-controlled trials	Expectation
27	Bingel et al. [42], 2011	Clinical trial	Treatment expectation on drug efficacy	Mitigating bias in sham-controlled trials	Expectation
28	Frisaldi et al. [43], 2017	Commentary article	Patients' Expectations in Clinical Trials	Mitigating bias in sham-controlled trials	Expectation
29	Dworkin et al. [45], 2010	Topical review	Placebo and treatment group responses in postherpetic neuralgia vs. painful diabetic peripheral neuropathy	Study population	Placebo
30	Freeman et al. [46] 2015	Cohort study	Predictors of placebo response in peripheral neuropathic pain	Study population	Placebo
31	Arakawa et al. [47], 2015	Systematic review and meta-analysis	Placebo Response in Clinical Trials in Neuropathic Pain	Study population	Placebo
32	Skyt et al. [48], 2015	Review	Placebo effects in chronic pain	Study population	Placebo
33	Niemansburg et al. [49], 2015	Review	Ethics of sham-controlled trials	Ethics	Risk-benefit balance, informed consent, deliberate deception
34	Miller et al. [50], 2004	Commentary article	Sham procedures and the ethics of clinical trials	Ethics	Risk-benefit balance
35	Freedman et al. [51] 1987	Commentary article	Equipoise and the ethics of clinical research.	Ethics	Equipoise
36	Horng et al. [53], 2003	Commentary article and proposed framework	Ethical framework for the use of sham procedures in clinical trials.	Ethics	Risk-benefit balance, informed consent, deliberate deception
37	Miller et al. [65], 2005	Review	Deception in research on the placebo effect	Ethics	Deliberate deception

### 3.1 Types of sham devices

The most cited types of sham devices are described.

#### 3.1.1 No perceivable output

A favoured method due to its simplicity in numerous device trials is to use the same device for both the active and sham arm but simply disconnect the sham device from its power source creating an inactive control [[12], [13], [14]]. Whilst this presents a simple solution, this type of sham fails to replicate often expected sensations or side effects associated with the active treatment. This can then jeopardise treatment credibility [[15], [16], [17]] and lead to unblinding [12]. To overcome this, recommendations suggest the exclusion of patients with previous experience of the intervention and avoiding cross-over designs [15,18].

#### 3.1.2 Subtherapeutic dosing

This can involve various methods such as shorter treatment duration and lower strength stimulation.

#### 3.1.3 Shorter treatment duration

This method has been used in numerous transcutaneous electrical nerve stimulation (TENS) trials where the sham device omits pulses for a couple of seconds before being shut off [19]. A critique of such trials is that even short-duration stimulation could be associated with albeit perhaps a smaller therapeutic effect [19].

#### 3.1.4 Lower strength stimulation

Stimulation at an intensity considered to be subtherapeutic has been used in various neuromodulation trials. For example, a recent neuromodulation trial evaluated the use of a restorative neurostimulator designed to restore multifidus neuromuscular control [20]. In this trial the sham device employed low-level stimulation eliciting a single muscle twitch (0.4 mA, 31 ms) compared to 30 min of contraction/relaxation active stimulation (20Hz and 214 ms). Although there were perceivable differences in both devices the participant's instructions were scripted to maintain blinding and all participants were told 'they may or may not perceive stimulation responses'. Following treatment all participants were asked to guess treatment allocation, within the control group 44% guessed treatment allocation correctly compared to 59% in the treatment group. These results suggest that the sham was perceived as credible and blinding was successfully maintained during this trial. Therefore low-level stimulation presents a useful option when considering sham controls providing information provided to participants is well considered and validity of blinding is assessed.

A recent systematic review of randomized sham‐controlled trials of spinal cord stimulation (SCS) describes four further studies that used lower-intensity tonic stimulation as sham control [21]. A highlighted limitation of these studies was the absence of pre-trial testing of the intended sham.

Without pre-testing of the sub-therapeutic shams, the absence of a true therapeutic benefit cannot be confidently excluded [21]. Therefore, there is an arguable need for research that explores the mechanism of action of the active device to construct a truly effective sham.

### 3.2 Design issues

The literature discusses various issues surrounding the design of a sham intervention.

#### 3.2.1 Mechanism of action

This issue was also highlighted by Template for Intervention Description and Replication (TIDieR) checklist and guide [22]. The report highlights that for too many sham-controlled trials the supposed mechanisms for the active treatment are unclear, and therefore it also remains unclear as to whether the proposed shams are truly ‘inactive’. They recommended that study methods should demonstrate an understanding of mechanisms of action and thereby which specific components of the ‘active’ arm need to be controlled [22]. Low-frequency nerve stimulation (1–2 Hz) to induce long-term synaptic depression (LTD) is one area where this has been explored. Animal and human studies have demonstrated that stimulation parameters such as stimulus duration, frequency and strength of stimulus are important to effectively induce LTD [[23], [24], [25]]. Based on results from these experimental studies one can understand which stimulation parameters will not induce LTD [26]. This highlights how experimental studies can inform clinical studies.

#### 3.2.2 Validation

To support the conduct of high-quality research the validity of the sham device should be determined before use [27]. There are several aspects to consider in terms of validation. For example, in the acupuncture literature, various non-penetrating sham techniques have been in use since the late 1990s [27]. These involve the use of a non-penetrating placebo needle, the needle tip of which simply presses against the skin and is concealed in an opaque guide tube that is indistinguishable from the active penetrating needles. Validation of these methods was performed through credibility testing on patients to ensure that the treatments could be blinded. Following this, these techniques became the gold standard sham intervention for numerous acupuncture trials [27]. A recent systematic review however highlighted that even such an inert seeming sham was associated with an actual effect [28]. They found that sham acupuncture techniques and 'real acupuncture' had similar effects on biomarkers and therefore that sham acupuncture was not inert [28]. This illustrates the necessity for validation to be supported by physiological evidence of no effect.

A pilot study by Sheffer et al. [29] looked specifically at the development of sham high-frequency repetitive transcranial magnetic stimulation (rTMS). The replication of a perceivable sensation by the sham device was considered an important factor to prevent unblinding. The group developed a sham that used focal stimulation of the scalp and used brain imaging to confirm that this stimulation was not associated with the physiological effects of cortical activation. Following patient evaluation and brain imaging, they concluded focal electrical stimulation can be an effective sham control for high-frequency rTMS. Studies that include patient evaluation in addition to physiological testing can be costly and require time and resources and are unsurprisingly rare [30].

### 3.3 Sham-controlled trials and bias

Bias refers to a type of error that affects how a result is interpreted due to the way the study was designed or conducted [31]. The literature describes various issues surrounding bias in sham-controlled trials.

#### 3.3.1 Blinding

The main justification for the use of a sham control is to facilitate the conduct of double-blinded trials [5,6]. Therefore, ensuring adequate blinding is particularly important to this type of study. However, bias associated with inadequate blinding of treatment allocation is cited as one of the major sources of bias in sham RCTs [8,32,33].

In certain cases, to ensure an intervention is delivered safely and accurately it may not be possible for the clinician delivering the treatment to be blinded to treatment allocation. For example, for surgical procedures, it would be necessary in most cases for the surgeon to be aware of the differences between active and sham treatments. It would also be hard to blind a clinician delivering treatment when there are evident differences in treatment response between sham and active treatments.

Equally patient blinding can be easily broken if patients become aware of potential differences between sham and active interventions. This can occur in cross-over trials after crossover of treatment arms or if patients communicate potential intervention differences to one another [34]. Researchers, therefore, need to consider the different ways in which this could occur. For example, ensuring patients from both treatment arms are not asked to wait in the same waiting areas. Or ensuring supporting information that may describe the active device (i.e., in the device manual or manufacturer's webpage) is adapted.

To limit detection bias in sham-controlled trials (bias associated with how outcomes are evaluated) it is strongly advised that independent, blinded assessors of outcome are involved [18,22]. Additionally, it is recommended that all double-blind trials adequately describe all measures used to blind participants and researchers to allow confident interpretation of the risk of unblinding bias within a given study [22,35]. However, it has been illustrated by various systematic reviews of sham-controlled studies that adequate description of study blinding is generally poor [19,21,36,37].

#### 3.3.2 Assessment of blinding

To ensure blinding has been successful studies need to consider how it is assessed [35,38]. An early review conducted by Hrobjartsson analysed a random sample of blinded randomized clinical trials indexed in The Cochrane Central Register of Controlled Trials [33]. Although this is not specific to sham-controlled trials they identified 1599 blinded trials and found only 31(2%) of those trials reported tests for the success of blinding. In most cases, the assessment of blinding was only conducted for patients, and they conclude that to demonstrate successful blinding, the assessment should include all individuals that are described as blinded (e.g., assessors of outcome). Furthermore, they highlight that there is also the uncertainty of the best way to assess blinding and a lack of formal measures to do this. Most studies ask people to guess between the experimental and sham and there is some debate as to whether an additional ‘don't know’ category should also be included [18,33]. Further variation exists concerning when to assess. A positive test conducted during, or after the end of, the sham-controlled trial, cannot be interpreted as a clear indication of bias, as ‘unblinding’ may be caused by the experience of a true treatment effect [33]. Assessment immediately after an intervention may provide information regarding the credibility of the sham however does not assess how blinding was maintained during the study. There is therefore huge potential for variation across studies in how assessment of blinding is conducted. Whilst there is no consensus on the optimal assessment methods studies need to incorporate a clear description of assessment and assessment results.

#### 3.3.3 Clinical interactions

Clinical interactions can lead to unblinding of subjects by clinicians, either consciously or subconsciously [[39], [40], [41]]. To mitigate this information relating to active and sham treatments needs to be delivered and presented in an equal and comparable way. TIDieR guides researchers concerning this and asks for studies to provide detailed documentation and reporting of key study elements such as patient monitoring, verbal and written instructions provided, who provides what, how, where, and when [22].

#### 3.3.4 Expectation

Bingel et al. in a study using functional magnetic resonance imaging found that positive and negative treatment expectation was related to the activation of different areas of the cortex. Positive expectancy was associated with activity within the endogenous opioid system and enhanced analgesic effect, and negative expectancy impacts the hippocampus and abolished analgesic response [42]. The power of expectation is especially significant for sham-controlled trials, as both patients and clinicians expect that half the sample will receive the sham intervention. It has therefore been proposed that patient expectation of benefit is assessed before they commence a trial, and that perception of effectiveness is assessed on trial completion [43,44]. An important implication of the above findings is the weight of verbal and nonverbal communication concerning expectation. This will involve considering not just what happens in the clinic/treatment room but also what can be communicated within waiting areas and via the web and social media concerning both active and sham devices.

### 3.4 Study population

Several randomized, double-blind clinical trials in neuropathic pain have failed to demonstrate a significant difference between active treatment and sham treatments, despite previous positive results of pre-clinical studies [45,46]. This has in part been attributed to variations in placebo responses between different types of neuropathic pain syndromes [45,47]. A systematic review by Arakawa considered variations in placebo responses in neuropathic pain syndromes [47]. They demonstrate that the proportion of patients expected to have a 50% or better pain reduction in placebo control groups can be hugely different depending on the type of neuropathic pain syndrome. For example, a response rate of 23% was reported for trials of peripheral neuropathic pain, 15% for posttraumatic peripheral neuropathic pain and 26% for painful diabetic peripheral neuropathy (95% CI) [47]. Additionally, within neuropathic pain syndromes, the presence of certain symptom characteristics can also influence the response rate (the number of patients that show a positive response). For instance, studies that include symptoms of hyperalgesia have been suggested to have among the largest placebo responses [48]. This highlights how variable individual responses can be, even within similar conditions and the need to carefully consider how diagnostically homogenous a population has to be to demonstrate treatment efficacy [15]. This is more challenging for conditions which do not have a well-accepted diagnostic criterion. To ensure sham-controlled studies are adequately powered researchers therefore need to understand the known placebo responses for RCTs within that specific study population.

### 3.5 Ethics

Ethical concerns were the most reviewed area relating to the conduct of sham trials [6,49,50].

#### 3.5.1 Equipoise

Equipoise relates to whether it is ethical to allow patients to have an inferior treatment (sham) if researchers know one arm (active) is superior. In a seminal paper in the New England Journal of Medicine, Benjamin Freedman proposed the concept of equipoise [51]. He stated that “the equipoise requirement is satisfied if there is genuine uncertainty within the expert medical community about the preferred treatment-not necessarily on the part of the individual investigator-about the preferred treatment”. For example, although clinicians may feel peripheral nerve stimulation (PENS) is beneficial for neuropathic pain, NICE guidelines [52] suggest there is currently insufficient evidence of efficacy to support its use, therefore a trial comparing PENS to sham PENS would be considered to have equipoise. Justification of equipoise is therefore an important determinate relative to the conduct of sham-controlled trials.

#### 3.5.2 Risk-benefit balance

Sham-controlled trials can be considered unethical because participants assigned to the control group have no prospect of benefit from the trial, yet they are exposed to all the risks of the sham intervention. Conversely, when the efficacy of an intervention is not established or is under question it could be argued there are clear benefits from being assigned to the sham control. The use of a sham intervention should therefore appraise potential risks and harms as part of a risk-benefit analysis [49,50]. The literature suggests that risk-benefit analysis should consider; 1) the risk has been minimized concerning the scientific question to be answered, 2) the risk is not excessive, and 3) the risk is justified by important knowledge to be gained [49,53]. Whilst a researcher will have an in-depth knowledge of the subject area that will help quantify risk, an ethics committee that must approve whether the risk is acceptable may not. Therefore, researchers need to provide adequate and clear information that allows an ethics committee to determine risk-benefit.

#### 3.5.3 Informed consent

It is suggested that participants within sham-controlled trials have a greater risk of not appreciating or understanding all the potential implications of sham control, which in turn compromises informed consent [49,53]. To satisfy informed consent sham-controlled studies need to ensure and evaluate participants' understanding of the sham intervention.

Ethicists have also highlighted that for informed consent participants additionally need to understand the potential placebo benefits that the sham device may offer [6]. This may additionally improve study recruitment [6]. This is salient when we consider the fear of not receiving treatment benefits through not being allocated the active treatment has been identified to adversely impact a patient's willingness to participate in a study [34,54,55].

#### 3.5.4 Deliberate deception

In sham controlled studies study subjects are led to believe the control could plausibly be the active treatment and therefore subjects are deliberately deceived to facilitate blinding and reduce bias [56]. Deliberate deception has been suggested can violate the principles of patient autonomy and may cause clinicians to feel moral discomfort [53]. Consequently, ethical frameworks suggest that to justify the use of deliberate deception the following requirements should be met; 1) deliberate deception is required to obtain valid data, 2) there is full disclosure to subjects regarding the use of deliberate deception, 3) subjects are aware they may receive a sham procedure 4) subjects are debriefed when the blind is broken [49,53]. Researchers, therefore, need an awareness of all these points and ensure they are demonstrated within research design and study protocols.

## 4 Discussion

In a world that requires increasing reassurances to implement and develop evidence-based treatments, researchers need to convince funders, governance frameworks such as ethics, and patients of treatment efficacy. This needs to be supported by well-designed and appropriately conducted trials. The inclusion of a sham control in a device trial can reduce bias by facilitating the conduct of double-blinded trials and therefore aid the conduct of high-quality research. This review identified major and subcategory themes that describe quality items which if considered could improve the conduct of future sham-controlled interventional pain trials.

### 4.1 Design issues

Although guidelines call for studies to adequately describe how sham treatments have been tested and developed [22,57], what is striking from the literature is that very few studies do [16,58,59]. Testing of sham interventions adds additional time and cost to the conduct of a study. If a new interventional device, device trial or sham device is developed it must conform to medical devices regulation policy. This will include consideration of UKCA (UK Conformity Assessed) or CE (European Conformity) marking and ensuring adequate indemnity insurance are in place. The most utilised form of sham controls in neurostimulation trials appears to be an active device that is disconnected from any power source and therefore produces no output. This design negates some of the processes such as CE marking just discussed, however, as highlighted in the review carries a high risk of unblinding [60]. Further design options such as lower dose or subthreshold stimulation, fall short when the mechanisms of action for the active treatment are not fully understood as possible treatment effects cannot be excluded. To overcome such issues future studies could consider and explore basic science and industry partnerships to develop valid and robust sham interventions. As part of this process, patient and public involvement are further recommended to improve research design and outcomes and ensure sham devices are developed that are deemed relevant and credible to all stakeholders [61].

#### 41.1 Sham-controlled trials and bias

Under this theme blinding was the predominant issue. Overall blinding was found to be one aspect of trial conduct that was typically found to be poorly described in published trials [32,33,36]. Unblinding due to perceivable differences between sham and active interventions was cited as one of the most common sources of unblinding. Several papers recommended that patients with previous experience of the intervention should be excluded, cross-over designs should be avoided and providing partial disclosure in terms of expected side effects of treatment should be considered [15,22]. Many trials published after such recommendations appear to have incorporated many of these suggestions. Although most studies explained the differences between the sham and active devices, few described how these differences were explained and understood by both patients and clinicians. Conversely, in clinical practice, patient education is well-recognised as an important aspect of any treatment procedure. Therefore, there seems to be some disparity as to what is acceptable for research practice and clinical practice. Additionally, a common critique by the identified literature was that there is much variation, ambiguity and little guidance in terms of how and when blinding is assessed [35,38]. A basic requirement of studies is to provide an adequate description of measures taken to maintain blinding and justify when and how this is assessed [21,22,38].

#### 4.1.2 Expectation

Whilst assessment of blinding is commonly recommended but poorly implemented, assessment of treatment expectations appears to be less commonly considered. Treatment expectation represents an important multifactorial covariant, which is associated with considerable ambiguity in terms of how and when is best to assess and measure its influence within RCTs [43,44]. Studies should include a minimum assessment of expectation relative to treatment allocation and treatment efficacy, pre and post-treatment, for patients but also clinicians.

#### 4.1.3 Study population

Responses to sham treatments vary considerably between and within different study populations [45]. Accurate estimates relative to potential placebo responses are required to inform study design in terms of power calculations and statically analysis. Systematic reviews highlight significant variation in placebo responses between different types of neuropathic pain [47] and further variation relative to different sensory characteristics [48]. Stratification of patients by sensory phenotype at least for pain populations has been suggested, which could improve treatment selection and outcomes by allowing for mechanistically informed treatment selection [62,63]. On this basis, stratification of patients by condition and additionally, sensory phenotype could also help in terms of understanding and evaluating placebo responses to inform study design.

#### 4.1.4 Ethics

Various frameworks have been developed to help guide researchers through salient ethical issues [53,64]. The consensus shared by the ethical literature surrounding the conduct of sham-controlled trials is not whether it is ethical to conduct a sham but rather consider whether conditions that make it ethical have been met.

## 5 Conclusion

Good quality sham-controlled trials are needed to support the efficacy of untested or unproven treatments. Currently, the methods used for sham-controlled trials are not always clearly described or considered which limits the quality and validity of findings. A holistic appreciation of the issues associated with sham-controlled studies is needed to conduct good-quality sham-controlled research studies. We highlight salient issues and provide recommendations for the conduct of future trials.

## Funding

This research did not receive any specific grant from funding agencies in the public, commercial, or not-for-profit sectors.

## Declaration of competing interest

The authors declare that they have no known competing financial interests or personal relationships that could have appeared to influence the work reported in this paper.
